# Language-Related Differences in Prenatal Depression Screening Uptake in the USA Midwest, 2019–2024

**DOI:** 10.1192/bjo.2026.12074

**Published:** 2026-08-06

**Authors:** Amanda Luff, Akaninyene Noah, Emily Malloy, Ravi Mishra, Veronica Fitzpatrick, Anne Rivelli

**Affiliations:** Department of Obstetrics and Gynecology, Wake Forest University School of Medicine, Winston-Salem, North Carolina, USA; Advocate Aurora Research Institute, Advocate Health, Milwaukee, Wisconsin, USA; Aurora Midwifery & Wellness Center, Advocate Health, Milwaukee, Wisconsin, USA; Department of Paediatrics, Wake Forest University School of Medicine, Winston-Salem, North Carolina, USA; Division of Neonatology, Department of Paediatrics, Advocate Illinois Masonic Medical Center, Advocate Health, Chicago, Illinois, USA; Department of Implementation Science, Wake Forest University School of Medicine, Winston-Salem, North Carolina, USA

**Keywords:** Electronic health records, observational study, perinatal psychiatry, quality improvement, patients

## Abstract

Prenatal depression is a substantial contributor to maternal morbidity, and screening is an entry point to psychiatric assessment and treatment during pregnancy. Following updated guidelines and quality metrics for prenatal depression screening, we evaluated whether screening uptake differed by preferred language within a large USA healthcare system. We used electronic health record data to identify a retrospective cohort of deliveries at or beyond 20 weeks gestation in 2019–2024. We used logistic regression with a language×year interaction to estimate the adjusted marginal probabilities of screening by language preference. Among 92 063 pregnancies (77 828 individuals), screening increased substantially over time, but increases differed across language groups (*p* < 0.001). In 2019, screening probabilities were similar (English 0.50, Spanish 0.51, another language 0.46). By 2024, probabilities diverged (English 0.82, Spanish 0.72, another language 0.65). Unequal screening uptake can systematically underidentify prenatal depression among patients with non-English-language preference, with implications for equitable access to psychiatric care.

Prenatal depression affects nearly two in five pregnancies in the USA and, when untreated, is associated with reduced engagement in prenatal care, preterm birth, postpartum depression and impaired child neurodevelopment.^1–3^ Perinatal depression is a leading contributor of maternal mortality, with death by suicide accounting for up to 20% of postpartum deaths.^4,5^ Because most mental health symptoms during pregnancy are first identified in obstetric settings, prenatal depression screening is an important pathway to further assessment, follow-up and referral for psychiatric evaluation and treatment.^6,7^ The American College of Obstetricians and Gynecologists first recommended universal prenatal depression screening in 2015, and reaffirmed this in 2018.^8,9^ In 2019, the National Committee for Quality Assurance added prenatal depression screening and follow-up as a quality measure.^10^ As systems expand screening through standardised workflows and performance measurement, equitable delivery is essential to avoid systematic underidentification of prenatal depression in groups facing barriers to care. Language is a known barrier to prenatal depression screening.^11^ This study evaluated the relationship between language preference and uptake of prenatal depression screening within a large, diverse USA healthcare system.

## Method

The retrospective study of electronic health record (EHR) data was conducted within a large, vertically integrated healthcare system in the USA Midwest. The cohort comprised pregnancies resulting in births at or beyond 20 weeks gestation between 1 July 2019 and 30 June 2024, with prenatal care initiation in the first or second trimester. Pregnancy outcomes before 20 weeks are not classified as births and are therefore inconsistently captured in the EHR. However, we did not condition on fetal viability or outcomes after 20 weeks, because very preterm birth or stillbirth is associated with elevated postpartum depression risk, warranting their inclusion in prenatal screening samples.^12,13^ We excluded pregnancies with missing variables of interest by using listwise deletion (<5% of the sample). We reported findings following the Strengthening the Reporting of Observational Studies in Epidemiology (STROBE) guidelines.^14^

Prenatal depression screening was determined by presence of a partial or completed depression screening instrument in the EHR during the prenatal period, most commonly the Patient Health Questionnaire-9. We used logistic generalised estimating equations clustered by patient to account for individuals contributing multiple pregnancies. Using an interaction term, we evaluated whether the association between language and screening varied by delivery year, adjusting for maternal age, race/ethnicity, insurance, parity, gestational age, singleton/multiple gestation and the state in which prenatal care occurred. We calculated and graphed marginal probabilities of receiving prenatal depression screening across years for each language group. All analyses were conducted using StataNow/MP for Windows (Stata Corp., College Staton, Texas, USA; www.stata.com).

The authors assert that all procedures contributing to this work comply with the ethical standards of the relevant national and institutional committees on human experimentation and with the Helsinki Declaration of 1975, as revised in 2013. All procedures involving human patients were approved by The Wake Forest School of Medicine Institutional Review Board (identifier IRB00129720), with a waiver of informed consent because of the retrospective method of the study.

## Results

Between 2019 and 2024, 92 063 eligible pregnancies among 77 828 individuals resulted in a delivery in this healthcare system (for sample characteristics, see Supplementary Table 1). Pregnant people reported 74 preferred languages (Supplementary Table 2); because most languages had small counts, we report language preference as English (94%), Spanish (3%) and another language (3%), to ensure stable estimation.

**Fig. 1:**
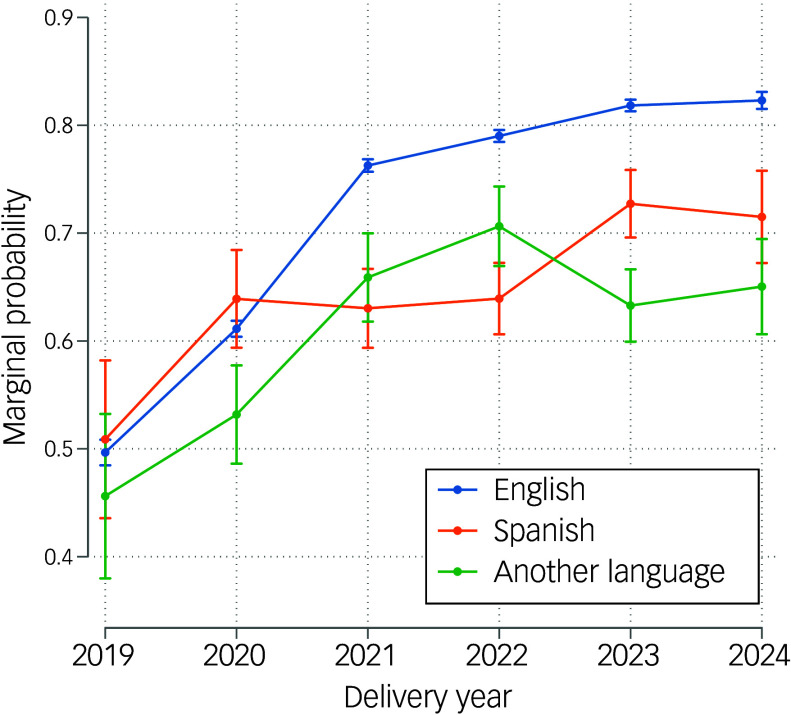
Marginal probabilities of prenatal depression screening by preferred language and delivery year, 2019–2024.

In the model estimating odds of prenatal depression screening, the interaction between language and year was statistically significant (*p* < 0.001). In 2019, marginal probabilities of prenatal depression screening were similar across groups: 0.50 (95% CI 0.48–0.51) for English, 0.51 (95% CI 0.44–0.58) for Spanish and 0.46 (95% CI, 0.38–0.53) for another language. Probabilities diverged over time (Fig. 1). By 2024, the probability of screening increased to 0.82 (95% CI 0.82–0.83) for English-language preference, 0.72 (95% CI 0.67–0.76) for Spanish-language preference and 0.65 (95% CI 0.61–0.69) for another language preference.

## Discussion

Prenatal depression screening increased from 2019 to 2024, but improvements were unequal across language groups, resulting in larger screening gaps by 2024 for individuals with non-English-language preference. Screening probabilities were similar across language groups early in the evaluation period, indicating that disparities emerged as screening expanded rather than reflecting persistent baseline differences. Prenatal depression screening is typically conducted in obstetric care as a primary pathway for identifying perinatal depression and connecting patients to mental health services.^6^ From a health-systems perspective, screening gaps represent missed opportunities for earlier intervention, including evidence-based psychotherapy and/or pharmacotherapy, care management and collaborative care. If screening is less consistently completed for people with non-English-language preference, fewer patients may be identified during routine prenatal care, potentially leading to these patients presenting with higher-acuity mental health conditions.

That disparities emerged as screening expanded suggests that implementation improved overall completion without fully addressing language access. Incorporation of screening into performance metrics may increase uptake, but without routine stratification by language preference, aggregate reporting can obscure widening inequities. Screening programmes often use standardised workflows, which may be built primarily for English-preference patients. When interpreter support or translated tools are needed, screening can require additional steps, such as locating validated translations and different documentation processes, increasing the burden for patients and clinicians. The presence of many preferred languages with small counts highlights a structural challenge to equitable screening, as validated instruments may be unavailable for some language groups. The Patient Health Questionnaire-9, the most commonly used depression screening tool, has official translations in over 50 languages, but not all have been validated.^15^ Additionally, depression screening is often administered through the patient portal in our system, which was available only in English until Spanish was added in 2023. Finally, small numbers across many languages can limit health system attention to any single group, even when the patient population with non-English-language preference is substantial.

Strengths of this study include the large cohort across multiple years within a diverse health system, and use of EHR-derived measures to evaluate real-world screening implementation. The analytic approach accounted for individuals contributing multiple pregnancies and examined whether the association between language and screening changed over time, which directly addresses how inequities may emerge during programme expansion. A limitation to this research is that screening was identified based on documentation of a screening instrument in the EHR; this may not fully capture whether screening was completed as intended, understood by the patient or administered with interpreter support when needed. Second, language preference recorded in the EHR may not perfectly reflect language proficiency, literacy or need for language support services, and the ‘another language’ category necessarily aggregates heterogeneous language groups. Third, this analysis evaluated receipt of screening rather than downstream steps such as severity of symptoms, follow-up, referral completion or treatment initiation; therefore, we cannot directly quantify how screening gaps translated into differences in mental health service access or outcomes. Finally, results reflect a single health system and may not generalise to settings with different screening workflows or language-access resources.

In summary, prenatal depression screening increased substantially from 2019 to 2024, yet language-related inequities widened as screening expanded. Given that mental health-related morbidity is a substantial contributor to maternal morbidity and that screening is a key entry point to mental health services, equitable screening implementation should be a priority for health systems and psychiatric care.^3,5^ Stratifying quality metrics by language preference and designing multilingual screening workflows may help ensure equitable screening expansion, promoting access to essential perinatal mental healthcare.

## Supporting information

10.1192/bjo.2026.12074.sm001 Luff et al. supplementary material Luff et al. supplementary material

## Data Availability

The data that support the findings of this study are available upon reasonable request from the corresponding author, A.L., subject to institutional data sharing procedures. The data are not publicly available to protect patient privacy. These results have been previously posted as a preprint on MedRxiv.^16^

## Supplementary material

The supplementary material is available online at https://doi.org/10.1192/bjo.2026.12074

## References

[1] Field T. Prenatal depression risk factors, developmental effects and interventions: a review. J Preg Child Health 2017; 4: 301. 28702506 10.4172/2376-127X.1000301 PMC5502770

[2] Sulley S , Adzrago D , Mamudu L , Odame EA , Atandoh PH , Tagoe I , et al. Assessment of prenatal depression among U.S. pregnant women without access to paid sick leave and regular place of care: National Health Interview Survey of U.S.-born and non-U.S.-born. Prevent Med Rep 2023; 35: 102322. 10.1016/j.pmedr.2023.102322 PMC10404555 37554349

[3] Jang M , Ramaiyer M , Olson S , Voegtline K , Esguerra C. Association between antepartum depressive symptoms and prenatal care utilization and milestones: a retrospective cohort study. BMC Pregnancy Childb 2025; 25: 392. 10.1186/s12884-025-07489-0 PMC11969695 40181309

[4] Lindahl V , Pearson JL , Colpe L. Prevalence of suicidality during pregnancy and the postpartum. Arch Womens Ment Health 2005; 8: 77–87. 15883651 10.1007/s00737-005-0080-1

[5] Lommerse KM , Mérelle S , Rietveld AL , Berkelmans G , Van Den Akker T. The Netherlands Audit Committee Maternal Mortality and Morbidity. The contribution of suicide to maternal mortality: a nationwide population-based cohort study. BJOG 2024; 131: 1392–8. 38344899 10.1111/1471-0528.17784

[6] Byatt N , Xu W , Levin LL , Moore Simas TA. Perinatal depression care pathway for obstetric settings. Int Rev Psychiatry 2019; 31: 210–28. 30701995 10.1080/09540261.2018.1534725

[7] Cox EQ , Sowa NA , Meltzer-Brody SE , Gaynes BN. The perinatal depression treatment cascade: baby steps toward improving outcomes. J Clin Psychiatry 2016; 77: 1189–200. 27780317 10.4088/JCP.15r10174

[8] American College of Obstetricians and Gynecologists. Committee Opinion No. 630: screening for perinatal depression. Obstetr Gynecol 2015; 125: 1268–71. 10.1097/01.AOG.0000465192.34779.dc 25932866

[9] American College of Obstetricians and Gynecologists. ACOG Committee Opinion No. 757: screening for perinatal depression. Obstet Gynecol 2018; 132: e208–12. 30629567 10.1097/AOG.0000000000002927

[10] National Committee for Quality Assurance. Results for Measures Leveraging Electronic Clinical Data for HEDIS . National Committee for Quality Assurance, 2021 (https://wpcdn.ncqa.org/www-prod/wp-content/uploads/2021/11/Special-Report-Reporting-Results-for-Measures-Leveraging-Electronic-Clinical-Data-for-HEDIS.pdf).

[11] Eakley R , Lyndon A. Disparities in screening and treatment patterns for depression and anxiety during pregnancy: an integrative review. J Midwifery Womens Health 2024; 69: 847–62. 39054664 10.1111/jmwh.13679 PMC11622364

[12] Pace CC , Spittle AJ , Molesworth CM-L , Lee KJ , Northam EA , Cheong JLY , et al. Evolution of depression and anxiety symptoms in parents of very preterm infants during the newborn period. JAMA Pediatr 2016; 170: 863. 27428766 10.1001/jamapediatrics.2016.0810

[13] Lewkowitz AK , Rosenbloom JI , Keller M , López JD , Macones GA , Olsen MA , et al. Association between stillbirth ≥23 weeks gestation and acute psychiatric illness within 1 year of delivery. Am J Obstetr Gynecol 2019; 221: 491.e1–22. 10.1016/j.ajog.2019.06.027 PMC6829063 31226297

[14] von Elm E , Altman DG , Egger M , Pocock SJ , Gøtzsche PC , Vandenbroucke JP. The Strengthening the Reporting of Observational Studies in Epidemiology (STROBE) statement: guidelines for reporting observational studies. J Clin Epidemiol 2008; 61: 344–9. 18313558 10.1016/j.jclinepi.2007.11.008

[15] Pfizer. Patient Health Questionnaire (PHQ) Screeners . Pfizer, 2026 (https://www.phqscreeners.com/select-screener).

[16] Luff A , Rivelli A , Akaninyene N , Malloy E , Mishra R , Fitzpatrick V. Language-related differences inprenatal depression screening uptake, US Midwest 2019-2024. Medrxiv [Preprint] 2026. Available from: 10.64898/2026.04.07.26350332. PMC13458377 42557900

