# Supplementary material for: Language-Related Differences in Prenatal Depression Screening Uptake in the USA Midwest, 2019–2024
Source: BJPsych Open. 2026 Aug 6;12(5):e208. doi: 10.1192/bjo.2026.12074 (PMC13458377; doi:10.1192/bjo.2026.12074)
Supplement: Supplementary file 1 [file S2056472426120742sup001.pdf]

Supplemental Table 1. Sample Characteristics

	n(%) or mean (SD)
**N (Pregnancies)**	92,063
**Prenatal Depression Screening**	
No	24,696 (26.8%)
Yes	67,367 (73.2%)
**Maternal Age**, mean(SD)	29.8 (5.3)
**Gestational Age at Birth**, mean(SD)	38.5 (2.3)
**Insurance Type**	
Private	60,168 (65.4%)
Public	29,745 (32.3%)
Other Insurance	947 (1.0%)
Self-Pay	1,203 (1.3%)
**Preferred Language**	
English	86,470 (93.9%)
Spanish	2,882 (3.1%)
Another Language	2,711 (2.9%)
**Parity**	
0	34,396 (37.4%)
1	30,788 (33.5%)
2	15,698 (17.1%)
3+	11,109 (12.1%)
**Singleton or Multiple Gestation**	
Singleton	89,252 (96.9%)
Multiple	2,811 (3.1%)
**State**	
Illinois	34,038 (37.0%)
Wisconsin	58,025 (63.0%)

Supplementary Table 2: Language Preferences of Prenatal Patients

Language	Count	Percent
**Individuals**	**77,829**	
**English**	72,839	93.59
**Spanish**	2689	3.46
**Arabic**	405	0.52
**Russian**	221	0.28
**Hmong**	191	0.25
**Ukrainian**	155	0.20
**Burmese**	121	0.16
**Karen**	119	0.15
**Rohingya**	118	0.15
**Vietnamese**	106	0.14
**Polish**	85	0.11
**Chinese**	58	0.07
**Gujarati**	56	0.07
**Urdu**	54	0.07
**Hindi**	53	0.07
**Swahili**	52	0.07
**Mongolian**	45	0.06
**French**	39	0.05
**Chin**	34	0.04
**Somali**	31	0.04
**Albanian**	29	0.04
**Korean**	22	0.03
**Nepali**	22	0.03
**Pashto**	22	0.03
**Punjabi**	19	0.02
**Amharic**	16	0.02
**Dari**	15	0.02
**Portuguese**	15	0.02
**Tigrinya**	14	0.02
**Telugu**	13	0.02
**Malay**	10	0.01
**Malayalam**	10	0.01
**Serbian**	10	0.01
**Tamil**	9	0.01
**Turkish**	9	0.01
**Assyrian**	8	0.01
**Japanese**	8	0.01
**Romanian**	8	0.01
**Bengali**	7	0.01
**K'iche**	7	0.01
**Uzbek**	7	0.01
**Lao**	6	0.01
**Lingala**	6	0.01
**Thai**	6	0.01
**Kayah**	5	0.01
**Kinyarwanda**	5	0.01
**Bulgari**	4	0.01
**Tagalog**	4	0.01
**Farsi**	3	0.00
**Oromo**	3	0.00
**Tajik**	3	0.00
**Yoruba**	3	0.00
**Akan**	2	0.00
**Greek**	2	0.00
**Italian**	2	0.00
**Karenni**	2	0.00
**Khmer**	2	0.00
**Kyrgyz**	2	0.00
**Wolof**	2	0.00
**Belarusian**	1	0.00
**Bosnian**	1	0.00
**Croatia**	1	0.00
**Dinka**	1	0.00
**Hebrew**	1	0.00
**Kosreaen**	1	0.00
**Kunama**	1	0.00
**Lithuanian**	1	0.00
**Marathi**	1	0.00
**Mon**	1	0.00
**Moroccan**	1	0.00
**Quechua**	1	0.00
**Slovak**	1	0.00
**Sorani**	1	0.00
**Sundanese**	1	0.00
